# Effect of Cathodic Protection on Reinforced Concrete with Fly Ash Using Electrochemical Noise

**DOI:** 10.3390/ma14092438

**Published:** 2021-05-07

**Authors:** Jorge García-Contreras, Citlalli Gaona-Tiburcio, Irene López-Cazares, Guillermo Sanchéz-Díaz, Juan Carlos Ibarra Castillo, Jesús Jáquez-Muñoz, Demetrio Nieves-Mendoza, Erick Maldonado-Bandala, Javier Olguín-Coca, Luis Daimir López-León, Facundo Almeraya-Calderón

**Affiliations:** 1 Universidad Autonoma de San Luis de Potosi, Facultad de Ingeniería, Av. Dr. Manuel Nava #8 Zona Universitaria, 78290 San Luis Potosí, Mexico; jorge.garcia@uaslp.mx (J.G.-C.); guillermo.sanchez@uaslp.mx (G.S.-D.); carlos.castillo@uaslp.mx (J.C.I.C.); 2 Universidad Autonoma de Nuevo Leon, FIME-Centro de Investigación e Innovación en Ingeniería Aeronáutica (CIIIA), Av., Universidad s/n, Ciudad Universitaria, 66455 San Nicolás de los Garza, Mexico; Jesus.jaquezmn@uanl.edu.mx; 3 Instituto Potosino de Investigación Científica y Tecnológica, IPICYT, División de Ciencias Ambientales, Camino a la Presa de San José 2055, Lomas 4 Sección, C.P., 78216 San Luis Potosí, Mexico; irene.lopez@ipicyt.edu.mx; 4 Universidad Veracruzana, Facultad de Ingeniería Civil, 91000 Xalapa, Mexico; dnieves@uv.mx (D.N.-M.); erimaldonado@uv.mx (E.M.-B.); 5 Universidad Autónoma del Estado de Hidalgo, Área Académica de Ingeniería y Arquitectura, Carretera Pachuca-Tulancingo Km. 4.5., 42082 Hidalgo, Mexico; olguinc@uaeh.edu.mx (J.O.-C.); luis_lopez@uaeh.edu.mx (L.D.L.-L.)

**Keywords:** electrochemical noise, fly ash, concrete, cathodic protection, microstructure

## Abstract

Corrosion of steel reinforcement is the major factor that limits the durability and serviceability performance of reinforced concrete structures. Impressed current cathodic protection (ICCP) is a widely used method to protect steel reinforcements against corrosion. This research aimed to study the effect of cathodic protection on reinforced concrete with fly ash using electrochemical noise (EN). Two types of reinforced concrete mixtures were manufactured; 100% Ordinary Portland Cement (OCP) and replacing 15% of cement using fly ash (OCPFA). The specimens were under-designed protected conditions (−1000 ≤ E ≤ −850 mV vs. Ag/AgCl) and cathodic overprotection (E < −1000 mV vs. Ag/AgCl) by impressed current, and specimens concrete were immersed in a 3.5 wt.% sodium chloride (NaCl) Solution. The analysis of electrochemical noise-time series showed that the mixtures microstructure influenced the corrosion process. Transients of uniform corrosion were observed in the specimens elaborated with (OPC), unlike those elaborated with (OPCFA). This phenomenon marked the difference in the concrete matrix’s hydration products, preventing Cl^−^ ions flow and showing passive current and potential transients in most specimens.

## 1. Introduction

In the construction industry, corrosion of steel reinforcement is one of the main issues. It is considered the leading cause of the premature deterioration of reinforced concrete structures [1,2,3]. The most predominant factors affecting the steel bars can be physical, mechanical, biological, or chemical [4,5]. Corrosion is the most frequent and relevant deterioration suffered by reinforced concrete structures, particularly in structures located in an aggressive marine environment [6,7]. Corrosion of reinforcement in the marine environment usually occurs because aggressive agents such as chloride ions are an electrochemical process [8,9].

The cement industry is one of the two largest producers of carbon dioxide (CO_2_), generating between 5–8% CO_2_ total emissions to the environment. In the future, the CO_2_ emissions are predicted around 10–15% [2,10]. The supplementary cementitious materials (SCM) are employed to reduce CO_2_ emissions and replace, in a portion, Portland cement [2,11,12,13,14,15].

New alkali-activated materials have been employed in place of OPC with a different type of ashes. The employ of different ashes showed good results increasing the durability of concrete. OPC has been replaced by ashes as slags furnace ash (SFA), metakaolin (MK), and fly ash (FA), among others [16,17,18]. SCM as sugar cane bagasse ash (SCBA) and rice husks ash (RHA) has been studied in the last 20 years to develop a better solution for reinforced structure [19,20,21,22]. Diverse research has been reported on the corrosion behavior of SCM and the structural, mechanical, chemical and structural properties [23,24,25]. The employ of SCM is an eco-friendly and cost-effective solution due to the natural properties of these materials [8,26]. An essential factor of SCM materials is their faster cure time in comparison with OPC.

Another critical factor of these SCM is that they cure faster than OPC, making them even more suitable for precast components.

Corrosion of reinforcing steel is the leading cause of deterioration in concrete. When steel corrodes, the resulting rust occupies a greater volume than the steel. The action of Cl^−^ ions are considered the most critical factor influencing the corrosion intensity of reinforced concrete [27,28,29]. The Cl^−^ ion causes the breakdown of the steel’s normal passive condition in concrete and corrosion development. An efficient method to control steel reinforcement corrosion in concrete structures is the impressed current cathodic protection (ICCP). ICCP is an efficient method to stop or control the steel reinforcement’s corrosion in concrete structures. ICCP is known for active or passive steel conditions; it is necessary to have specific conditions for both pH and potential in the electrochemical system [27,28,29,30,31,32,33]. On the other hand, studies reported that the fly ash particles react with the calcium hydroxide, generating hydration products that strongly affect decreasing the concretes porosity. Also, mechanical properties are increased when using these ashes and blast furnace slag, given the increasing volume of C-S-H gel [32].

Electrochemical noise (EN) technique for corrosion applications has allowed many advances in recent years interesting for corrosion science. A particular advantage of EN measurements is detecting and analyzing the early stages of localized corrosion.

Electrochemical noise describes the spontaneous low-level potential and current fluctuations occurring during a corrosion process. During the corrosion process, predominantly electrochemical cathodic and anodic reactions can cause small transients in electrical charges on the electrode. These transients manifest in potential and current noise exploited in a corrosion map [34,35]. The rupture and re-passivation of passive films are associated with the stochastic process (anodic and cathodic transients), as well as the formation and pitting propagation are related to the deterministic process [36,37].

Transients are linked to anodic and cathodic reactions because of stochastic processes (rupture and re-passivation of the passive film) and deterministic processes (formation and propagation of pitting) [36,37]. The time-series of potential or current provides helpful information about the corrosion process.

Ma, et al. [38,39] indicate that EN data is influenced by the measurement mode, the surface area of the working electrodes, the electrolytic resistance and the symmetry of the electrode system. Xia, et al. [40] mention several mathematical methods and parameters that can analyze EN data to identify corrosion form and corrosion rates. They are classified into three groups: the time domain, the frequency domain, and the time-frequency domain. The statistical analysis it includes parameters as noise resistance (Rn), Skewness, Kurtosis, localization index (*LI*) Chaos analysis, Recurrence quantification analysis and Fractal analysis. The Fast Fourier Transform includes power spectral density, noise impedance, etc; and time-frequency domains method; have the analysis Hilbert-Huang transform, Discrete Wavelet transform, Stockwell transform and others) [41,42,43,44,45,46,47]. *LI*, skewness, and kurtosis values have been related to different corrosion types and the asymmetry of the distribution of EN data [47,48,49,50,51,52].

This research aimed to study the Effect of cathodic protection on reinforced concrete with fly ash using electrochemical noise. Two types of reinforced concrete mixtures were elaborated: 100% Ordinary Portland Cement (OCP) and replacing 15% of cement using fly ash (OCPFA). The specimens were under-designed protected conditions (−1000 ≤ E ≤ −850 mV vs. Ag/AgCl) and cathodic overprotection (E < −1000 mV vs. Ag/AgCl) by impressed current, and specimens concrete were immersed in a 3.5 wt.% sodium chloride (NaCl) Solution. This concentration was used to simulate an aggressive environment that contains chlorides where civil works can be built based on reinforced concrete.

## 2. Materials and Methods

### 2.1. Reinforced Concrete Specimens

This research were designed for eigth specimens: OCP and FA concretes mixtures according to NMX-C-414-ONNCCE [53] of 30 MPa at 28 days. The Fly Ash (FA)—Class F type addition was according to ASTM C618-94a [54], obtained from a coal-fired power plant in Coahuila, Mexico. The Research group has tested supplementary materials as a partial substitute cement and has found good results with fly ash [2,12,19,22,23,27]. Therefore, in this study Fly Ash was used as a partial substitute for OPC with a replacement percentage of 15%. Table 1 shows the chemical composition of both materials used obtained by X-ray fluorescence (XRF) analysis provided by the supplier.

The dosage of concrete mixtures was carried out according to the method of ACI 211.1 [55]. This method is based on the physical properties of coarse and fine aggregates, see Table 2.

The OPC and FA mixtures were made with a ratio of water/cement of 0.66. The specimens were cylindrical with dimensions of 15 high by 7.5 cm in diameter [53]. In all the specimens, AISI 1018 Carbon Steel bars were embedded. The steel bars dimensions were 9.5 mm in diameter and 10.5 cm in length. The curing of all specimens was carried out by immersion in water for 28 days, according to NMX-C-159 standard [56].

### 2.2. System of Protection and Cathodic Overprotection

**Figure 1:**
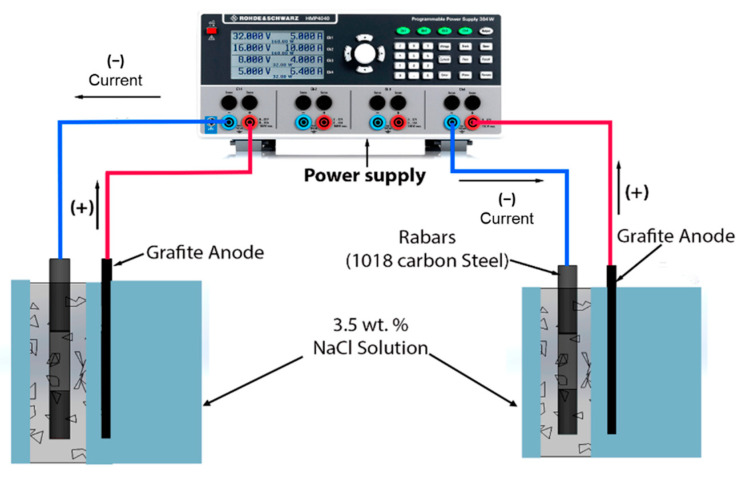
Experimental system to maintain the specimens under protection and cathodic overprotection.

After the curing step, the specimens of both mixtures were immersed in a 3.5 wt.% NaCl solution and an impressed current cathodic protection system were induced. Anode, ¼ in. graphite bars with 0.635 cm. in diameter and a length of 15 cm. were used. The capacity of the employed rectifier was 20 V/10 A. Four of the specimens were treated to a potential protection criterion (−1000 mV ≤ E ≤ −850 mV vs. Ag/AgCl), and the rest of them with overprotection (E < −1000 mV vs. Ag/AgCl) [7]. The protection and overprotection levels were consistently maintained by an experimental system, as shown in Figure 1. The nomenclature used considers the following: 2–3 = months of exposure, FA = Fly ash, P = Protected, OP = overprotected.

### 2.3. Electrochemical Noise Test

The electrochemical noise (EN) technique was used to evaluate 100% Ordinary Portland Cement (OCP) and the other with replacing 15% of cement using fly ash (OCPFA), immersed in 3.5 wt.% in NaCl solutions. The EN measurements were carried out using ACM Instruments (Manchester, UK)—Gill-AC potentiostat/galvanostat/ZRA (Zero Resistance Ammeter).

The EN experiments were carried out in based to ASTM G199-09 standard to determine the noise resistance (*R_n_*) and corrosion rate. To each experiment was employed two nominally identical specimens as the working electrodes (WE1 and WE2) and a saturated calomel electrode as the reference electrode (RE) [51,57]. The electrochemical current noise (ECN) was measured with a galvanic coupling current between two identical working electrodes. The electrochemical potential noise (EPN) was measured linking one of the working electrodes and the reference electrode. The current and potential electrochemical noise was monitored concerning each electrode’s electrolyte combination under open-circuit conditions. 1024 data points were obtained with a scanning rate of 1 data per second. The current and potential electrochemical noise was monitored concerning each electrode’s electrolyte combination under open-circuit conditions. 1024 data points were obtained with a scanning rate of 1 data per second [51,52].

DC trend signal was removed from the original EN signal by the polynomial method, from signal without DC statistical data (*R_n_*, Kurtosis, and skewness) was obtained from the signal without DC. For PSD (power spectral density) analysis, a Hann window was applied before being transformed to the FFT frequency domain (fast Fourier transform). Data analysis was processed with a program made in MATLAB 2018a software (Math Works, Natick, MA, USA).

### 2.4. Scanning Electron Microscopy (SEM)

Scanning electron microscopy (SEM, Jeol JSM 5610LV, Tokyo, Japan) was as well utilized for concrete surface morphology analyzed to different magnifications, operating at 20 kV, WD = 10 mm. The chemical composition of these concrete specimens was obtained by energy dispersive X-ray spectroscopy (EDS, EDAX, Tokyo, Japan).

## 3. Results

### 3.1. Microstructure of OPC and OPCFA by Scanning Electron Microscopy.

**Figure 2:**
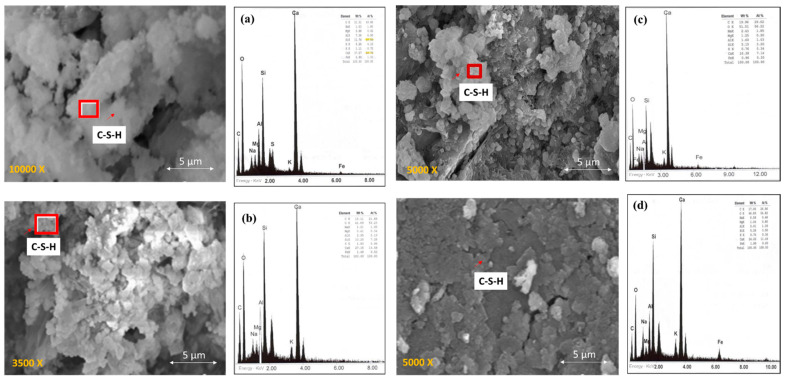
SEM surface morphology micrographs and elemental analysis by X-ray dispersive energy spectroscopy (EDS) of specimens made with the OPC mixture: (**a**) 2OPC-P Ca/Si = 2.24, (**b**) 2OPC-OP Ca/Si = 1.86, (**c**) 3OPC-P Ca/Si = 2.23, (**d**) 3OPC-OP Ca/Si = 3.25.

Figure 2 shows the microstructure of specimens made with the mixture of OPC. In the 2OPC specimen (Figure 2a), it is possible to visualize the hydration products in the form of cotton. However, the elemental analysis by X-ray dispersive energy spectroscopy (EDS) shows that the Ca/Si ratio is 2.24, which is within the range of a concrete porous [58,59].

The specimen 2OPC-OP (Figure 2b) shows that the concrete obtained higher hydration than protected. The hydration occurs because the Ca/Si ratio is 1.86, which is in the range of well-hydrated concrete. According to the EDS analysis, a higher amount of hydrated calcium silicates (C-S-H gel) [27,32,60].

For specimens with three months of exposure (Figure 2c,d), it is observed that the microstructure of the concrete is more porous than those exposed to two months. Porous could be attributed to Ca/Si ratios in intervals of 2.23 and 3.25, which is typical of porous concrete. With very few hydration products (C-S-H gel) shown by the images and reinforced by the EDS analysis.

**Figure 3:**
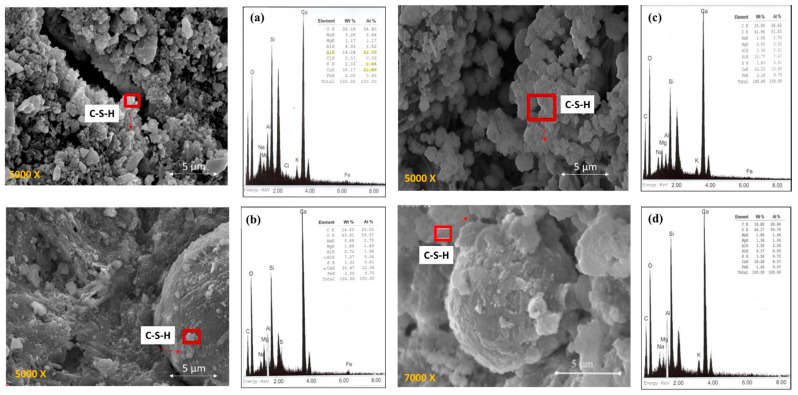
SEM surface morphology micrographs and elemental analysis by X-ray dispersive energy spectroscopy (EDS) of specimens made with the OPCFA mixture: (**a**) 2OPCFA-P Ca/Si = 1.78, (**b**) 2OPCFA-OP Ca/Si = 2.45, (**c**) 3OPCFA-P Ca/Si = 1.44, (**d**) 3OPCFA-OP Ca/Si = 1.40.

Figure 3 shows the microstructure of the specimens made with the mixture with OPCFA. In Figure 3a,b, the microstructure of the specimen was exposed to two months under protection and overprotection conditions. The 2OPCFA-P specimen shows an appreciable amount of hydration products (C-S-H gel). According to the EDS analysis, the Ca/Si ratio is within a hydrated concrete range with a value of 1.78. As for specimen 2OPCFA-OP, it is observed that the Ca/Si ratio increased to a value of 2.45, indicating greater porosity but with fewer hydration products in this specimen. In the specimens at three months of exposure with both protective conditions, they show the effects of fly ash particles, generating more hydration products. In Figure 3c,d reinforced by the EDS analysis with the Ca/Si ratios results, which tends to decrease with time in this type of mixtures, depending on the hydration time and the amount of fly ash in the mixture.

### 3.2. Electrochemical Noise

EN signal is composed of random, stationary, and DC variables. It is necessary to separate DC from random and stationary components to analyze EN data because DC creates false frequencies and interferes in visual, statistical, and PSD analysis. In this way, when DC is removed, corrosion data presented at low frequencies are conserved [51]. EN can be represented by Equation (1) [43,44,45]:

(1) $$x\left(t\right)=m_{t}+s_{t}+Y_{t}$$

where *x*(*t*) is the EN time series, *m_t_*, is the DC component, *s_t_* is the random component, and *Y_t_* is a stationary component. The latter two are the functions that define the corrosion system [36]. The polynomial method, as defined in Equation (2), defines noise signal (*x_n_*) and polynomial of “*n*” grade (*p_o_*) at n-th term (*a_i_*) in “*n*” time to obtain a signal without trend (*y_n_*) [33,42,43,44]:

(2) $$y_{n}=x_{n}-\overset{p_{o}}{\underset{i=0}{\sum }}a_{i}n^{i}$$

**Figure 4:**
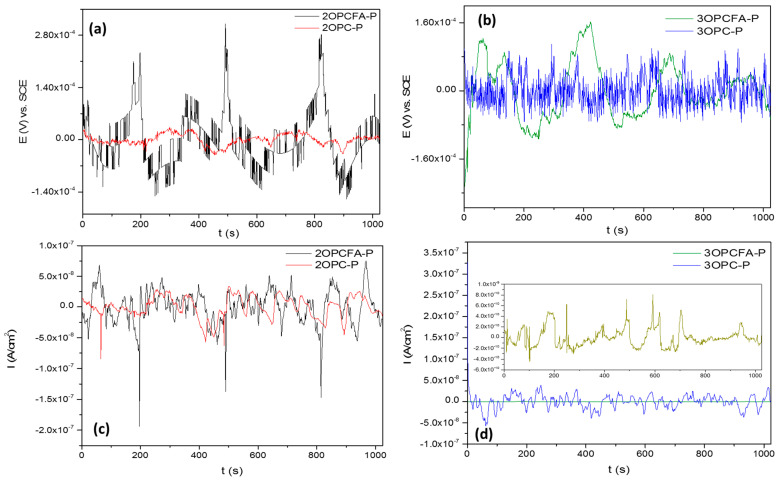
Electrochemical noise-time series for specimens subjected to Impressed current cathodic protection of reinforced concrete mixtures (OPC-P and OPCFA-P). (**a**,**b**) ENP, 2 and 3 months, (**c**,**d**) ENC, 2 and 3 months.

Figure 4 shows the EN signal for specimens subjected to cathodic protection current. The EPN signal of OPC-P samples exposure two months (Figure 4a,b) presents potential transients of 3 × 10^−4^ V maximum. Low potentials are associated with high stability produced by oxidation-reduction events. The ECN signal corroborated it with low amplitude transients and fluctuations of ×10^−7^ (A/cm^2^) order. The behavior of EPN and ECN signals is related to the uniform corrosion process. In the case of FA samples, the potential transients show individual events of low frequency. On the other hand, OPC-P samples presented a higher instability associated with the passive layer’s break. When concrete contains FA and the passive layer is broken, it will be regenerated easier than samples with only OPC.

The sample of OPC-P exposed three months (Figure 4c,d) presented similar behaviors in EPN and ECN signals, associated with uniform corrosion. However, the sample with FA presented current values of ×10^−10^ (A/cm^2^) amplitude. The low current value indicates passivation in the sample after three months of exposure [13]. The decrease of current demand suggests that the passive layers become more stable with the exposure. Also, at three months, EPN and ECN signals did not present the anodic transients that samples with two months of exposure presented.

**Figure 5:**
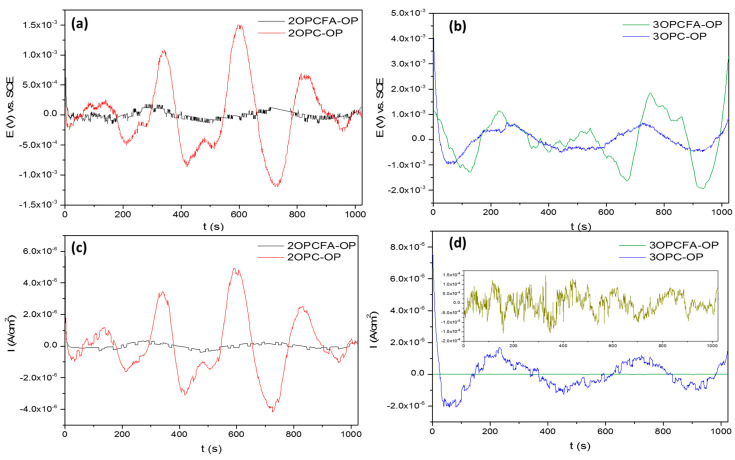
Electrochemical noise-time series for specimens subjected to Impressed current cathodic protection of reinforced concrete mixtures (OPC-OP and OPCFA-OP). (**a**,**b**) ENP, 2 and 3 months, (**c**,**d**) ENC, 2 and 3 months.

Figure 5 shows the EN signal of samples subjected to cathodic overprotection current. For overprotected samples, the current and potential values are low frequencies and amplitudes. However, the variation of fluctuations is high, associated with an unstable system. The FA samples showed the same behavior, with low amplitude and signals but with high variations; this could provoke the system’s instability. Also, samples with the only OPC presented higher current demand indicating a higher corrosion kinetic. The sample 2OPCFA-OP presented potential and current transients of very short frequencies and very low amplitudes. This behavior was presented due to stable passivation and ionic diffusion through this passive film.

The three months samples with OPC presented potential and current transients characteristic of unstable uniform corrosion, with low frequency and low amplitude behaviors. However, the decrease of frequency presented at two months of exposure is related to a higher harmful activity. The samples with FA presented lower current demand related to lower corrosion kinetic and suggest a passivity phenomenon.

#### 3.2.1. Statistical Analysis

Electrochemical noise resistance (*R_n_*) is determined by standard deviation from time series values. These statistical values give corrosion kinetics information. Cottis and Turgoose [50] found a relationship between the increase of variance and standard deviation with an increase in corrosion rate. The standard deviation (S) of the current or potential is calculated using the relationship.

(3) $$\sigma_{x}=\sqrt{\bar{x^{2}}}=\sqrt{\frac{1}{N}\overset{N}{\underset{i=1}{\sum }}{\left(x_{1}-\bar{x}\right)}^{2}}$$

where *x*_1_ is the transient ECN or transient EPN, $\bar{x}$ the average ECN or average EPN, and n is the number of pints in the recording.

Noise resistance (*R_n_*) is defined as the ratio of the standard deviation of potential to the standard deviation of current: (Equation (4)).

(4) $$R_{n}=\frac{\sigma_{v}}{\sigma_{I}}*A$$

*R_n_* and *R_p_* are equivalent to the Stern–Geary equation (Equation (5) can be applied as an analog relation to determining corrosion. *R_p_* is expressed in Ω·cm^2^, *i_corr_* is the corrosion current density, and B in V. is a constant resulting from a combination of the anodic and cathodic Tafel slopes. Recommended values of B are 0.026 and 0.052 V for active and passive systems, respectively [61].

(5) $$R_{n}=\frac{Β}{i_{corr}}=\frac{b_{a}b_{c}}{2.303\;\left(b_{a}+b_{c}\right)\cdot i_{corr}}$$

Some authors related the EN signal to statistical analysis with the metal surface to the corrosion process [51]. The *I_r.m.s_* is obtained by Equation (6), where *X* is the average of EN data, *n* the data number, and *σ* the standard deviation:

(6) $$r.m.s=\sqrt{Xn^{2}+\sigma^{2}}$$

The localization index (*LI*) is defined as the ratio of the standard deviation of ECN, $\sigma_{i}$, and *I_r.m.s_* (root mean square of the noise current) (Equation (7)).

(7) $$LI=\frac{\sigma_{i}}{I_{r.m.s}}$$

Values obtained can be associated with the system is corrosion type [51,52,62]. This research will take more parameters to determine the corrosion type (Table 3).

This research employed Kurtosis and skewness to try to define the corrosion type. Localization index (*LI*) was not considered because Mansfeld and Sun [63] in 1995 concluded that *LI* can present limitations and should be used with discretion. In 2001, Reid and Eden [64] developed a patent where they identified corrosion type based on statistical moments with skewness and Kurtosis (Equations (8) and (9)), which are the 3rd and 4th statistical moments [51,63,64]:

(8) $$skewness=\frac{1}{N}\overset{N}{\underset{i=1}{\sum }}\frac{{\left(x_{i}-\bar{x}\right)}^{3}}{\sigma^{3}}$$

(9) $$kurtosis=\frac{1}{N}\overset{N}{\underset{i=1}{\sum }}\frac{{\left(x_{i}-\bar{x}\right)}^{4}}{\sigma^{4}}$$

Statistical calculations have a standard error (*SE*) that generates uncertainty in the results. The following Equation can be provided, where *N* is the number of data studied [65]. Hence, when the data number is significant, the standard error will be lower than when the data number is high.

(10) $$SE=\sqrt{\frac{24}{N}}$$

*SE* is 0.153; values obtained will take *SE* as a parameter of uncertainty. Corrosion type determined by Kurtosis and skewness is shown in Table 4:

Kurtosis and skewness were applied to the ECN signal to determine the corrosion mechanism based on corrosion kinetic. Table 5 shows *R_n_*, *i_corr_*, skewness, and Kurtosis from EN signal filtered with a 9th-grade polynomial to remove DC signal. For protected samples, *LI* indicates values near to 1; this value is related to a disordered system and high variation of standard deviation. For that reason, it is essential to analyze with more precise methods as Skewness and Kurtosis in potential. Employing skewness and analyze the protected samples presented uniform corrosion.

The overprotected samples presented high values of *LI*, skewness, and Kurtosis. High *LI*, skewness, and Kurtosis values are associated with the high current variation with low current and potential values; any variation can change the results. The samples with FA presented minor variation; this could be attributed to a passive process.

#### 3.2.2. Power Spectral Density PSD Analysis

For Power spectral density PSD analysis, it is necessary to transform the time-domain EN to frequency-domain by applying FFT. There is a correlation with EN signal (with a polynomial filter applied), after which spectral density is calculated with Equations (11) and (12) [66,67].

(11) $$R_{xx}\left(m\right)=\frac{1}{N}\overset{N-m-1}{\underset{n=0}{\sum }}x\left(n\right)\cdot x\left(n+m\right),\;\;\mathrm{when}\;\mathrm{values}\;\mathrm{are}\;\mathrm{from}\;0<m<N$$

(12) $$\psi_{x}\left(k\right)=\frac{\gamma \cdot t_{m}}{N}\cdot \overset{N}{\underset{n=1}{\sum }}\left(x_{n}-{\bar{x}}_{n}\right)\cdot e^{\frac{-2\pi kn^{2}}{N}}$$

For PSD interpretation, it is necessary to evaluate the slope and the frequency zero limits (*ψ*^0^). The cut frequency indicates the slope begins. The slope could be helpful to find the corrosion mechanism [49]. The slope is defined by *β_x_* and is represented by Equation (13):

(13) $$log\Psi_{x}=-\beta_{x}\log f$$

The frequency zero limits (*ψ*^0^) give material dissolution information because PSD is related to the total energy present in the system [37]. It is essential to clarify that material dissolution is only present in the current PSD [48,65,68]. The following table was proposed by Mansfeld et al. [69] in 1998 to determine the corrosion phenomena occurring on the material surface; this table is adapted to decibels (see Table 6) [70].

**Figure 6:**
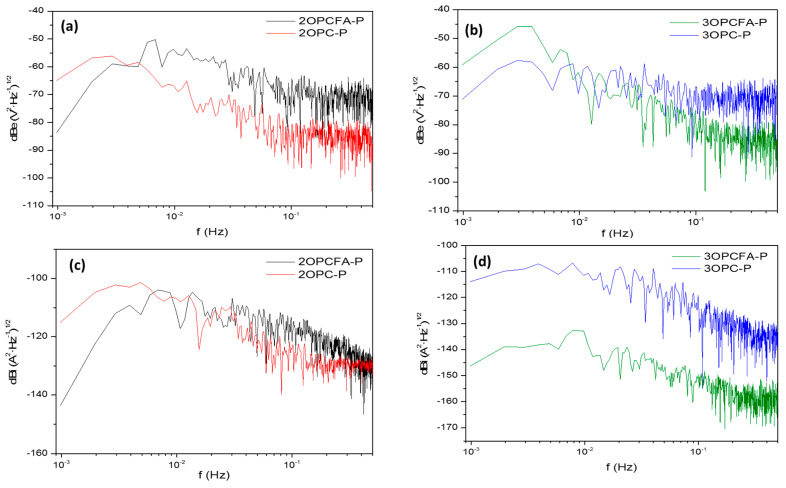
Power spectral density (PSD) for specimens subjected to Impressed current cathodic protection of reinforced concrete mixtures (OPC-P and OPCFA-P). (**a**,**b**) Potential, 2 and 3 months, (**c**,**d**) Current, 2 and 3 months.

Figure 6 shows the PSD results; Figure 6a,b presents PSD for EPN and ECN. Samples have values of slope in potential related to uniform corrosion (see Table 7). The *ψ*^0^ values showed that 2OPC-P presents a faster degradation related to a high corrosion kinetic. With samples exposed three months, the behavior is the same that the exposed two months. Also, samples exposed three months showed higher *ψ*^0^ values indicating that the materials will be dissolved faster (see Figure 6c,d).

**Figure 7:**
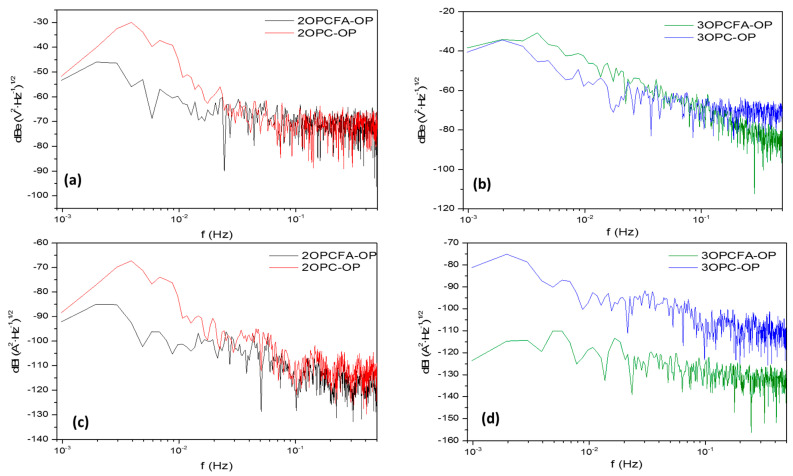
Power spectral density (PSD) for specimens subjected to Impressed current cathodic protection of reinforced concrete mixtures (OPC-OP and OPCFA-OP). (**a**,**b**) ENP, 2 and 3 months, (**c**,**d**) ENC, 2 and 3 months.

The overprotected samples exposed two months presented slops related to uniform corrosion (Figure 7a,b). 2OPC-OP presents fluctuations at high frequencies even in current or potential; this indicates instability in the processes. The samples exposed for three months present values related to uniform corrosion. Only the sample with FA presented a passivation system’s slope value (−23 dB(V)). The material dissolution is higher for OPC samples, and FA mixed showed higher resistance to degradation. Also, overprotected samples present a higher *ψ*^0^ value, indicating higher corrosion kinetic (see Table 7). Values of *R_n_* and *ψ*^0^ presented relation, with a high Rn a low *ψ*^0^ corresponds. Type and corrosion mechanisms presented some uncertainty for slope analysis in PSD [67,70]. For that reason, slope values present limitations to determine the type of corrosion in reinforced concrete mixtures.

## 4. Discussion

The concrete microstructure for the OPC mixture shows high values in the Ca/Si ratio, being porous concrete. Except for the specimen 2OPCO-P with a ratio of 1.86, the microstructure does not differ in the corrosion process since four specimens indicate uniform corrosion. This behavior indicates that the oxide layer was formed after two months of the steel surface and was maintained both exposure times. Corrosion can be caused because Cl^−^ ion diffuses more quickly through the mixtures microstructure, regardless of protection current and cathodic overprotection. Specimens with FA have a different behavior due to their microstructure and there Ca/Si ratio. FA’s Ca/Si ratio shows a better hydrated and less porous concrete, which prevents Cl^−^ ions. Koleva et al. mention that for a high Ca/Si ratio in values of 2.19–2.95, they counteract the positive aspects of a low porosity in the concrete matrix.

The results showed the benefits of employ SCM with OPC. Rubi [71] comments that it is possible to tailor cement with specific properties from the mechanical point of view and durability and chemical stability in highly aggressive environments.

Li and Hou [72,73] study the influence of fly ash in complex cement pastes. The results of Liu and Hou showed that the addition of fly ash means a decrease in the cement content when using cementitious materials, which leads to a decrease in the content of Ca(OH)_2_ in complex pastes. The cure time of these materials indicates that the crystal of Ca(OH)_2_ still exists in complex binder pastes at the age of 360 days. With the extension of the curing ages, a large amount of low CaO/SiO_2_ ratio of C-S-H gel is generated, which causes complex binder pastes to gradually compact.

Fly ash is a pozzolanic material. It is a finely divided amorphous alumino-silicate with varying amounts of calcium. Calcium mixed with Portland cement and water will react with the calcium hydroxide The hydration of Portland cement releases various calcium-silicate hydrates (C-S-H) and calcium-aluminate hydrates. These pozzolanic reactions are beneficial to the concrete. Pozzolanic reactions increase the quantity of the cementitious binder phase (C-S-H). To a lesser extent, calcium-aluminate hydrates improve the long-term strength and reduce the system’s permeability [74,75,76].

The fly ash is calcium content is perhaps the best indicator of how the fly ash will behave in concrete [77]. However, other compounds such as alkalis (Na_2_O and K_2_O), carbon, and sulfate (SO_3_) can also affect fly ash performance.

Electrochemical noise transients are related to different types of corrosion processes. The pitting process consists of high insensitivity transients with a high repetition rate. If the behavior is mixed, there are transients and oscillations of short amplitude. The uniform process is related to oscillations of low amplitude from the pattern noise. EPN and ECN signals of specimens subjected to cathodic protection current is related to uniform corrosion process show transients low frequency. OPC-P samples presented a higher instability associated with the break of the passive layer. When concrete contains FA and the passive layer is broken, it will be regenerated easier than OPC samples. The EN signal for overprotected samples at 2 and 3 months has current and potential values with low frequencies and amplitudes. Variation of fluctuations is high, associated with an unstable system. Samples with FA presented lower current demand related to lower corrosion kinetic and suggest a passivity phenomenon.

It has been determined that *R_n_* is indeed equivalent to the polarization resistance [43]. Therefore, the 1/*R_n_* values are proportional to the corrosion rate according to Ohm’s law and the Stern-Geary Equation [61]. The *R_n_* in OPC samples at 2 and 3 months have low values, which indicates a higher corrosion rate. The overprotection FA samples at three months have high Rn values, representing a better behavior in the corrosion kinetics.

In [78], a study about Steel bars embedded in concrete without fly ash and fly ash (the content of 20%) was tested under complete immersion in a 3.5 wt.% NaCl solution. Monitoring of OCP, LRP, and EIS was used to evaluate the steel bars corrosion behavior. The results obtained from electrochemical tests show that partial replacement of fly ash has enhanced corrosion resistance. Also, reduced corrosion rate due to the decrease of permeability to chloride ions. The results obtained from electrochemical noise in OPC and OCPFA samples with cathodic protection complement these investigations to improve the corrosion resistance of steel.

Studies of reinforced concrete applying protection methods focus on adding corrosion inhibitors or rehabilitating structures by electrochemical extraction of chlorine ions or realkalination of concrete. The application of cathodic protection current to reinforced concrete has been commonly studied by electrochemical techniques such as OCP, PP, or EIS. However, EN is not used.

Chloride-induced localized corrosion of embedded steel has been widely known for many years to be one of the most dangerous corrosion types in reinforced concrete [79,80,81]. Cathodic protection and supplementary materials such as fly ash decrease the effects of corrosion in reinforced concrete produced in harsh environments. Koleva [82,83,84] investigated the applicability and efficiency of an alternative for ICCP for reinforced concrete based on pulse technology. In specimens made with OPC exposed in 5 wt.% NaCl for a time of 460 days. CP pulse efficiency was evaluated using conventional monitoring techniques (half-cell potential and polarization decay) and electrochemical techniques (potentiodynamic polarization and electrochemical impedance spectroscopy). Recorded parameters even suggest the pulse CP regimens better performance compared to the conventional CP technique.

Using impressed current cathodic protection is essential to define the protection levels to obtain good results and differentiate the effects of cathodic protection and supplementary materials. In this study, the protected and overprotected OPCFA samples have good corrosion resistance. Lu et al. [85] investigated the efficacy of active and passive protection of the the pre-corroded steel reinforcement (1%, 3%, and 6% theoretical mass losses) in reinforced concrete (RC) columns using an externally bonded Carbon Fiber Reinforced Polymer (CFRP) Wrap. Active protection is a novel technique used to prevent corrosion achieved using the CFRP envelope as the anode of the ICCP. Electrochemical measurements were OCP, LPR, and EIS. The results indicate that active protection efficiency is closely related to the pre-corrosion of the protected specimens. Additionally, the efficiency of active protection decreased over time, which can be attributed to the CFRP anode’s deterioration and the CFRP/concrete interface. Passive protection was proved to be effective in all cases.

An ICCP system can promote hydrogen activity at the concrete-steel interface. The consumption of electrons from the corrosion process, so safe CP limits, must be established to prevent the risk of phenomena such as hydrogen-embrittlement of steel reinforcements or the concrete-steel interface degradation [27,86,87,88,89]. These positional variations in the cathodic current density make it difficult to maintain the same protection level throughout the RCS. In an ICCP system with a low applied current, oxygen and potential levels may be depleted, allowing water reduction to take place:

2H_2_O + 2e^−^ → H_2_ + 2OH^−^

Moreover, the initiation of fracture can be caused by molecular hydrogen absorbed on the steel surface.

Research with similar additions of fly ash and cathodic protection was carried out by Garcia et al. [27]. Garcia presented results on the adhesion between concrete-steel using the EIS test. Garcia founded that potassium, sodium, and hydrogen ion migration towards the concrete-steel interface play an essential role in bond loss between steel and concrete in structures under cathodic protection impressed current method. Due to a calcium hydroxide reaction, potassium ion content is lower in concrete specimens with fly ash. In contrast, sodium ion migration is higher in concrete containing fly ash due to the sodium ions small size. At the overprotection level, the potassium and sodium ions migrate more quickly than at the protection level. Hydrogen content is higher at the overprotection level than at protection. This phenomenon is attributed to the generation of molecular hydrogen at the overprotection level.

Garces [90] Study of the corrosion level of reinforcing steel bars embedded in Portland cement mortars containing different fly ash types. Concluding the influence of the type of fly ash to establish the durability of the concrete-steel system.

These results demonstrate the using supplementary cementitious materials in OPC increases the corrosion resistance when exposed to chlorides. These results would significantly contribute to creating a more sustainable concrete industry.

## 5. Conclusions

After studying the Effect of cathodic protection on reinforced concrete with fly ash using electrochemical noise.

- SEM and EDS observations indicated that the microstructures could be observed the hydration products (C-S-H gel) in both mixtures. Hydration influences some results on the Ca/Si ratio obtained, showing that the OPCFA mixtures had better hydration, indicating fewer pores in its microstructure.
- Results indicated that electrochemical noise tests have marked the difference the two mixtures microstructure, obtaining a uniform corrosion process for the specimens elaborated with the OPC mixture. Microstructure facilitated Cl^−^ ions to the steel surface. Contrary to the mixture made with OPCFA where most of the specimens showed passivity.
- After trend removal, EN signals conserved transients and fluctuation behavior and gave practical corrosion information removing DC.
- EN results show that Rn and *Ψ*^0^ parameters should be considered as a counterpart to calculate corrosion resistance of reinforced concrete mixtures.
- Overprotected samples presented less corrosion resistance than protected samples. This behaviour could be to steel embrittlement by hydrogen adsorption.
- Samples with FA presented a higher trend to passivation than samples with OPC. Besides, FA samples showed higher corrosion resistance.
- Statistical and PSD results showed that uniform corrosion predominates the systems. Although current values are shallow, the variation is considerable, and complicated the analysis for current signals, as potential is more stable than current, makes statistical and PSD analysis easier.
- The current protection and cathodic overprotection did not indicate some tendency in both mixtures behavior, for these exposure times exposure and ratio w/c.
- The discordance of statistical results could be related to developing a different corrosion process on the surface when LI indicates localized corrosion and skewness uniform corrosion. It suggests that localized and uniform corrosion occurs on the surface, but uniform corrosion is the predominant system.

## Figures and Tables

**Table 1:** Chemical composition of OPC and FA obtained by XRF.

Material	Compounds (wt.%)									
	CaO	SiO_2_	Al_2_O_3_	Fe_2_O_3_	MgO	SO_4_^2−^	Na_2_O	K_2_O	MnO	TiO_2_
Ordinary Portland Cement (CPC)	65.31	18.47	4.13	3.80	1.42	4.64	0.46	1.13	0.19	0.29
Fly Ash (FA)	3.26	57.3	28.14	5.21	0.56	0.32	0.51	1.52	-	1.21

**Table 2:** Dosage of conventional concrete and sustainable concrete, (kg/m^3^ of concrete, ratio w/c = 0.66).

Mixture	Fly Ash kg/m^3^	Aggregate Coarse kg/m^3^	Aggregate Fine kg/m^3^	Cement kg/m^3^	Water kg/m^3^
OPC	-	1049	781	310	205
OPCFA	46.5	1049	781	263.5	205

**Table 3:** Correlation between *LI* and type of corrosion expected [60].

Corrosion Type	*LI* (Value Range)
Localized	1.0–0.1
Mixed	0.1–0.01
Uniform	0.01–0.001

**Table 4:** Corrosion types evaluated by Kurtosis and skewness [65].

Corrosion Type	Potential		Current	
	Skewness	Kurtosis	Skewness	Kurtosis
Uniform	<±1	<3	<±1	<3
Pitting	<−2	>>3	>±2	>>3
Transgranular (SCC)	4	20	−4	20
Intergranular (SCC #1)	−6.6	18 to 114	1.5 to 3.2	6.4 to 15.6
Intergranular (SCC #2)	−2 to −6	5 to 45	3 to 6	10 to 60

**Table 5:** EN statistical parameters of specimens subjected to Impressed current cathodic protection of reinforced concrete mixtures (OPC-P/OP and OPCFA-P/OP).

Sample	*R_n_* (Ω·cm^2^)	*i_corr_* (mA/cm^2^)	*LI*	Corrosion Type	Skewness (Pot)	Corrosion Type	Kurtosis (Pot)	Corrosion Type
**Protected**								
2OPCFA-P	2779.05	0.0095557	1	Localized	−0.82	Uniform	6.7	Pitting
2OPC-P	826.78	0.31447	1	Localized	−0.65	Uniform	2.96	Pitting
3OPCFA-P	383777.68	0.0000677	1	Localized	0.19	Uniform	2.89	Uniform
3OPC-P	1830.34	0.0142	1	Localized	0.38	Uniform	2.81	Uniform
**Overprotected**								
2OPCFA-OP	404.08	0.0643422	0.49	Localized	−1.77	Pitting	14.24	Pitting
2OPC-OP	296.74	0.876967	0.93	Localized	0.46	Uniform	3.11	Pitting
3OPCFA-OP	177951.805	0.0001461	1	Localized	0.09	Uniform	2.93	Uniform
3OPC-OP	493.7	0.0526635	1	Localized	1.9	Pitting	14	Pitting

**Table 6:** *β* intervals to indicate the type of corrosion [70].

Corrosion Type	dB(V)·Decade^−1^		dB(A)·Decade^−1^	
	Minimum	Maximum	Minimum	Maximum
Uniform	0	−7	0	−7
Pitting	−20	−25	−7	−14
Passive	−15	−25	−1	1

**Table 7:** Parameters obtained by PSD for reinforced concrete mixtures (OPC-P/OP and OPCFA-P/OP).

Sample	*ψ*^0^ (dBi)	Β (dB [V])
2OPCFA-P	−143.716496	−7
2OPC-P	−64.8956506	−8
3OPCFA-P	−146.383815	−12
3OPC-P	−113.95617	−2
2OPCF-OP	−118.723889	−2
2OPC-OP	−122.621082	−3
3OPCFA-OP	−123.658499	−23
3OPC-OP	−81.2552579	−8

## Data Availability

The data presented in this study are available on request from the corresponding author.

## References

[1] Dacuan C.N., Abellana V.Y. (2021). Bond Deterioration of Corroded-Damaged Reinforced Concrete Structures Exposed to Severe Aggressive Marine Environment. Int. J. Corros..

[2] Erick Maldonado-Bandala E., Higueredo-Moctezuma N., Nieves-Mendoza D., Gaona-Tiburcio C., Zambrano-Robledo P.O., Hernández-Martínez H., Almeraya-Calderón F. (2020). Corrosion Behavior of AISI 1018 Carbon Steel in Localized Repairs of Mortars with Alkaline Cements and Engineered Cementitious Composites. Materials.

[3] Tapan M., Aboutaha R. (2011). Effect of steel corrosion and loss of concrete cover on strength of deteriorated RC columns. Constr. Build. Mater..

[4] Azad A.K., Ahmad S., Azher S.A. (2007). Residual strength of corrosion-damaged reinforced concrete beams. ACI Mater. J..

[5] Tondolo F. (2015). Bond behaviour with reinforcement corrosion. Constr. Build. Mater..

[6] Bilcik J., Holly I. (2013). Effect of reinforcement corrosion on bond behaviou. Procedia Eng..

[7] Ouglova A., Berthaud Y., Foct F., François M., Ragueneau F., Petre-Lazar I. (2008). The influence of corrosion on bond properties between concrete and reinforcement in concrete structure. Mater. Struct..

[8] Zhang X., Liang X., Wang Z., Huang H., Zhou H. An experimental study on effect of steel corrosion on the bondslip performance of reinforced concrete. Proceedings of the 5th International Conference on Durability of Concrete Structures.

[9] Mahasenan N., Smith S., Humphreys K. (2003). The cement industry and global climate change current and potential future cement industry CO_2_ emissions. Greenhouse Gas Control Technologies, Proceedings of the 6th International Conference, Kyopto, Japan, 1–4 October 2002.

[10] Gartner E. (2004). Industrially interesting approaches to “low-CO_2_” cements. Cem. Concr. Res..

[11] Wang J., Wu X.L., Wang J.X., Liu C.Z., Lai Y.M., Hong Z.M., Zheng J.P. (2012). Hydrothermal synthesis and characterization of alkali-activated slag–fly ash–metakaolin cementitious materials. Microporous Mesoporous Mater..

[12] Baltazar M.A., Bastidas D.M., Santiago G., Mendoza J.M., Gaona C., Bastidas J.M., Almeraya F. (2019). Effect of silica fume and fly ash admixtures on the corrosion behavior of AISI 304 embedded in concrete exposed in 3.5% NaCl solution. Materials.

[13] Ariza-Figueroa H.A., Bosch J., Baltazar-Zamora M.A., Croche R., Santiago-Hurtado G., Landa-Ruiz L., Mendoza-Rangel J.M., Bastidas J.M., Almeraya-Calderón F., Bastidas D.M. (2020). Corrosion Behavior of AISI 304 Stainless Steelreinforcements in SCBA-SF Ternary Ecological 3 Concrete exposed to MgSO_4_. Materials.

[14] Criado M., Bastidas D.M., Fajardo S., Fernández-Jiménez A., Bastidas J.M. (2011). Corrosion behaviour of a new low-nickel stainless steel embedded in activated fly ash mortars. Cem. Concr. Compos..

[15] Troconis de Rincón O., Montenegro J.C., Vera R., Carvajal A.M., De Gutiérrez R.M., Del Vasto S., Saborio E., Torres-Acosta A., Pérez-Quiroz J., Martínez-Madrid M. (2016). Reinforced Concrete Durability in Marine Environments DURACON Project: Long-Term Exposure. Corrosion.

[16] Santiago G., Baltazar M.A., Galván R., López L., Zapata F., Zambrano P., Gaona C., Almeraya F. (2016). Electrochemical Evaluation of Reinforcement Concrete Exposed to Soil Type SP Contaminated with Sulphates. Int. J. Electrochem. Sci..

[17] Talha Junaid M., Kayali O., Khennane A., Black J. (2015). A mix design procedure for low calcium alkali activated fly ash-based concretes. Constr. Build. Mater..

[18] Habert G., d’Espinose de Lacaillerie J.B., Roussel N. (2011). An environmental evaluation of geopolymer based concrete production: Reviewing current research trends. J. Clean. Prod..

[19] Maldonado-Bandala E., Jiménez-Quero V., Olguin-Coca J., Lizarraga L.G., Baltazar-Zamora M.A., Ortiz-C A., Almeraya C.F., Zambrano R.P., Gaona-Tiburcio C. (2011). Electrochemical Characterization of Modified Concretes with Sugar Cane Bagasse Ash. Int. J. Electrochem. Sci..

[20] Padhi R., Mukharjee B. (2017). Effect of rice husk ash on compressive strength of recycled aggregate concrete. J. Basic Appl. Eng. Res..

[21] Baltazar M.A., Mendoza J.M., Croche R., Gaona C., Hernández C., López L., Olguín F., Almeraya F. (2019). Corrosion behavior of galvanized steel embedded in concrete exposed to soil type MH contaminated with chlorides. Front. Mater..

[22] Pellegrini Cervantes J.M., Barrios Durstewitz P.C., Núñez Jaquez R., Almeraya Calderón F., Rodríguez-Rodríguez M., Fajardo-San-Miguel G., Martinez-Villafañe A. (2015). Accelerated corrosion test in mortars of plastic consistency with replacement of rice husk ash and nano-SiO_2_. Int. J. Electrochem. Sci..

[23] Pellegrini-Cervantes M.J., Almeraya-Calderon F., Borunda-Terrazas A., Bautista-Margulis R.G., Chacón-Nava J.G., Fajardo-San-Miguel G., Almaral-Sanchez J.L., Barrios-Durstewitz C., Martinez-Villafañe A. (2013). Corrosion Resistance, Porosity and Strength of lended Portland Cement Mortar Containing Rice Husk Ash and Nano-SiO_2_. Int. J. Electrochem. Sci..

[24] Saraswathy V., Song H.W. (2007). Corrosion performance of rice husk ash blended concrete. Constr. Build. Mater..

[25] Abu Bakar B.H., Putrajaya R., Abdulaziz H. (2010). Malaysian rice husk ash–improving the durability and corrosion resistance of concrete: Pre-review. Concr. Res. Lett..

[26] Hossain M.M., Karim M.R., Hasan M., Hossain M.K., Zain M.F.M. (2016). Durability of mortar and concrete made up of pozzolans as a partial replacement of cement: A review. Constr. Build. Mater..

[27] García J., Almeraya-Calderon F., Barrios C., Nuñez R., Lopez M., Rodríguez M., Martínez A., Bastidas J.M. (2012). Effect of cathodic protection on steel–concrete bond strength using ion migration measurements. Cem. Concr. Compos..

[28] Corral H.R., Arredondo R.S.P., Neri F.M., Gómez S.J.M., Almeraya C.F., Castorena G.J.H., Almaral S.J. (2011). Sulfate attack and reinforcement corrosion in concrete with recycled concrete aggregates and supplementary cementing materials. Int. J. Electrochem. Sci..

[29] Corral-Higuera R., Arredondo-Rea P., Neri-Flores M.A., Gómez-Soberón J.M., Almaral-Sánchez J.L., Castorena-González J.C., Almeraya-Calderón F. (2011). Chloride ion penetrability and Corrosion Behavior of Steel in Concrete with Sustainability Characteristics. Int. J. Electrochem. Sci..

[30] Erdoğdu S., Kondratovab L., Bremner T. (2004). Determination of chloride diffusion coefficient of concrete using open-circuit potential measurements. Cem. Concr. Res..

[31] Wyatt B.S. (1993). Cathodic protection of steel in concrete. Corros. Sci..

[32] Qingnan Zhana E., Abbas Z., Tang L. (2018). Predicting degradation of the anode–concrete interface for impressed current cathodic protection in concrete. Constr. Build. Mater..

[33] Pedeferri P. (1996). Cathodic protection and cathodic prevention. Constr. Build. Mater..

[34] Almeraya-Calderón F., Estupiñán F., Zambrano R.P., Martínez-Villafañe A., Borunda T.A., Colás O.R., Gaona-Tiburcio C. (2012). Análisis de los transitorios de ruido electroquímico para aceros inoxidables 316 y -DUPLEX 2205 en NaCl y FeCl. Rev. Metal..

[35] Mehdipour M., Naderi R., Markhali B.P. (2014). Electrochemical study of effect of the concentration of azole derivatives on corrosion behavior of stainless steel in H_2_SO_4_. Prog. Org. Coat..

[36] Kelly R.G., Scully J.R., Shoesmith D.W., Buchheit G. (2002). Electrochemical Techniques in Corrosion Science and Engineering.

[37] Kearns J.R., Eden D.A., Yaffe M.R., Fahey J.V., Reichert D.L., Silverman D.C., Kearns J.R., Scully J.R., Roberge P.R., Reirchert D.L., Dawson L. (1996). ASTM Standardization of Electrochemical Noise Measurement. Electrochemical Noise Measurement for Corrosion Applications.

[38] Ma C., Song S., Gao Z., Wang J., Hu W., Behnamian Y., Xia D.H. (2017). Electrochemical noise monitoring of the atmospheric corrosion of steels: Identifying corrosion form using wavelet analysis. Corros. Eng. Sci. Technol..

[39] Ma C., Wang Z., Behnamian Y., Gao Z., Wu Z., Qin Z., Xia D.H. (2019). Measuring atmospheric corrosion with electrochemical noise: A review of contemporary methods. Measurement.

[40] Xia D.H., Song S., Behnamian Y., Hu W., Cheng F., Luo J.L., Huet F. (2020). Review—Electrochemical Noise Applied in Corrosion Science: Theoretical and Mathematical Models towards Quantitative Analysis. J. Electrochem. Soc..

[41] Botana P.J., Bárcena M.M., Villero Á.A. (2002). Ruido Electroquímico: Métodos de Análisis.

[42] Gaona-Tiburcio C., Aguilar L.M.R., Zambrano-Robledo P., Estupiñán-López F., Cabral-Miramontes J.A., Nieves-Mendoza D., Castillo-González E., Almeraya-Calderón F. (2014). Electrochemical Noise Analysis of Nickel Based Superalloys in Acid Solutions. Int. J. Electrochem. Sci..

[43] Montoya-Rangel M., de Garza-Montes O.N., Gaona-Tiburcio C., Colás R., Cabral-Miramontes J., Nieves-Mendoza D., Maldonado-Bandala E., Chacón-Nava J., Almeraya-Calderón F. (2020). Electrochemical noise measurements of advanced high-strength steels in different solutions. Metals.

[44] Monticelli C. (1992). Evaluation of Corrosion Inhibitors by Electrochemical Noise Analysis. J. Electrochem. Soc..

[45] Park C.J., Kwon H.S. (2005). Electrochemical noise analysis of localized corrosion of duplex stainless steel aged at 475 °C. Mater. Chem. Phys..

[46] Suresh G.U., Kamachi M.S. (2014). Electrochemical Noise Analysis of Pitting Corrosion of Type 304L Stainless Steel. Corrosion.

[47] Cabral-Miramontes J.A., Barceinas-Sánchez J.D.O., Poblano-Salas C.A., Pedraza-Basulto G.K., Nieves-Mendoza D., Zambrano-Robledo P.C., Almeraya-Calderón F., Chacón-Nava J.G. (2013). Corrosion Behavior of AISI 409Nb Stainless Steel Manufactured by Powder Metallurgy Exposed in H_2_SO_4_ and NaCl Solutions. Int. J. Electrochem. Sci..

[48] Nagiub A.M. (2017). Electrochemical Noise Analysis for Different Green Corrosion Inhibitors for Copper Exposed to Chloride Media. Port. Electrochim. Acta.

[49] Dawson D.L., Kearns J.R., Scully J.R., Roberge P.R., Reirchert D.L., Dawson L. (1996). Electrochemical Noise Measurement: The definitive In-Situ Technique for Corrosion Applications?. Electrochemical Noise Measurement for Corrosion Applications STP 1277.

[50] Cottis R., Turgoose S., Mendoza-Flores J., Kearns J.R., Scully J.R., Roberge P.R., Reirchert D.L., Dawson L. (1996). The Effects of Solution Resistance on Electrochemical Noise Resistance Measurements: A Theorical Analysis. Electrochemical Noise Measurement for Corrosion Applications STP 1277.

[51] Jáquez-Muñoz J.M., Gaona-Tiburcio C., Cabral-Miramontes J., Nieves-Mendoza D., Maldonado-Bandala E., Olguín-Coca J., López-León L.D., Flores-De los Rios J.P., Almeraya-Calderón F. (2021). Electrochemical Noise Analysis of the Corrosion of Titanium Alloys in NaCl and H_2_SO_4_ Solutions. Metals.

[52] Lara-Banda M., Gaona-Tiburcio C., Zambrano-Robledo P., Delgado-E M., Cabral-Miramontes J.A., Nieves-Mendoza D., Maldonado-Bandala E., Estupiñan-López F., Chacón-Nava J.G., Almeraya-Calderón F. (2020). Alternative to Nitric Acid Passivation of 15-5 and 17-4PH Stainless Steel Using Electrochemical Techniques. Materials.

[53] (2014). NMX-C-414-ONNCCE-2014–Industria de la Construcción—Cementantes Hidráulicos - Especificaciones y Métodos de Ensayo.

[54] ASTM (2008). Standard Specification for Coal Fly Ash and Raw or Calcined Natural Pozzolan for Use in Concrete.

[55] ACI (2004). ACI 211.1-2004 Standard, Standard Practice for Selecting Proportions for Normal, Heavyweight, and Mass Concrete.

[56] ONNCCE (2004). NMX-C-159-ONNCCE-2004, Industria de la Construcción—Concreto—Elaboración y Curado de Especímenes en el Laboratorio.

[57] ASTM (2009). Standard Guide for Electrochemical Noise Measurement.

[58] Song G. (2000). Equivalent circuit model for AC electrochemical impedance spectroscopy of concrete. Cem. Concr. Res..

[59] Neville A. (1996). Properties of the Concrete.

[60] Morgan J.H. (1993). Cathodic Protection.

[61] Stern M., Geary A.L. (1957). Electrochemical polarization. I. A theoretical analysis of the shape of the polarization curves. J. Electrochem. Soc..

[62] Eden D.A., John D.G., Dawson J.L. (1997). Corrosion Monitoring. International Patent.

[63] Mansfeld F., Sun Z. (1999). Technical Note: Localization Index Obtained from Electrochemical Noise Analysis. Corrosion.

[64] Reid S.A., Eden D.A. (2001). Assessment of Corrosion. http://www.khdesign.co.uk/Patents/US6264824.Eden%20AI.pdf.

[65] Cottis R. (2001). Interpretation of Electrochemical Noise Data. Corrosion.

[66] Homborg A.M., Tinga T., Zhang X., Van Westing E.P.M., Ferrari G.M., Wit J.H.W., Mol J.M.W. (2017). A Critical Appraisal of the Interpretation of Electrochemical Noise for Corrosion Studies. Corrosion.

[67] Kelekanjeri V.S.K.G., Moss L.K., Gerhardt R.A., Ilavsky J. (2009). Quantification of the coarsening kinetics of γ’ precipitates in Waspaloy microstructures with different pri-or homogenizing treatments. Acta Mater..

[68] Legat A., Dolecek V. (1995). Corrosion Monitoring System Based on Measurement and Analysis of electrochemical Noise. Corrosion.

[69] Lee C.C., Mansfeld F. (1998). Analysis of electrochemical noise data for a passive system in the frequency domain. Corros. Sci..

[70] Homborg A.M., Cottis R.A., Mol J.M.C. (2016). An integrated approach in the time, frequency and time-frequency domain for the identification of corrosion using electrochemical noise. Electrochim. Acta.

[71] Mejia de Gutiérrez R. (2003). Effect of supplementary cementing materials on the concrete corrosion control. Rev. Metal..

[72] Li X., Yan J., Huaquan Yang H. (2015). Influence of Fly Ash on Microstructure of Complex Binder Pastes. Adv. Mater. Res..

[73] Hou J., Mei F., Mei Z. (2013). Quantitative Research on Strength Characteristics of Phosphogypsum Consolidation Materials. Appl. Mech. Mater..

[74] Marceau M.L., Gajda J., VanGeem M.G. (2002). Use of Fly Ash in Concrete: Normal and High Volume Ranges.

[75] Helmuth R. (1987). Fly Ash in Cement and Concrete.

[76] Malhotra V.M., Zhang M.-H., Read P.H., Ryell J. (2002). Long term mechanical properties and durability characteristics of high strength/high-performance concrete incorporating supplementary cementing materials under outdoor exposure conditions. ACI Mater. J..

[77] Thomas M.D.A., Shehata M., Shashiprakash S.G. (1999). The Use of Fly Ash in Concrete: Classification by Composition. Cem. Concr. Aggreg..

[78] Yoon-Seok C., Jung-Gu K., Kwang-Myong L. (2006). Corrosion behavior of steel bar embedded in fly ash concrete. Corros. Sci..

[79] Alvarez G.M., Galvele R.J. (1984). The mechanism of pitting of high purity iron in NaCl solutions. Corros. Sci..

[80] Feliu V., Gonzalez J.A., Andrade C., Feliu S. (1998). Equivalent circuit for modelling the steel-concrete interface. I. experimental evidence and theoretical predictions. Corros. Sci..

[81] Andrade C. (1993). Calculation of chloride diffusion coefficients in concrete from ionic migration measurements. Cem. Concr. Res..

[82] Koleva D.A., Guo Z., Breugel K., de Wit J.H. (2009). Conventional and pulse cathodic protection of reinforced concrete: Electrochemical behavior of the steel reinforcement after corrosion and protection. Mater. Corros..

[83] Koleva D.A., Hu J., Fraaij A., Beek H. Corrosion and Prevention 2005. Proceedings of the Conference Australian Corrosion Association, CAP05.

[84] Koleva D.A., Breugel K.W., de Wit J.H., Westing E., Copuroglu O., Fraaij L.A. (2008). Correlation of microstructure, electrical properties and electrochemical phenomena in reinforced mortar. Breakdown to multi-phase interface structures. Part II: Pore network, electrical properties and electrochemical response. Mater. Charact..

[85] Lu Y., Hu J., Li S., Tang W. (2018). Active and Passive Protection of Steel Reinforcement in Concrete Column Using Carbon Fibre Reinforced Polymer against Corrosion. Electrochim. Acta.

[86] Zampronio M.A., Fassini F.D., Miranda P.E.V. (1995). Design of ion-implanted hydrogen contamination barrier layers for steel. Surf. Coat. Technol..

[87] Chang J.J. (2002). A study of the bond degradation of rebar due to cathodic protection current. Cem. Concr. Res..

[88] Chang J.J., Yeih W., Huang R. (1999). Degradation of the bond strength between rebar and concrete due to the impressed cathodic current. J. Mater. Sci. Tech..

[89] Rasheeduzzafar M.G.A., Alsulaimani G.J. (1993). Degradation of bond between reinforcing steel and concrete due to cathodic protection current. ACI Mater. J..

[90] Garcés P., Andión L.G., Zornoza E., Bonilla M., Payá J. (2010). The effect of processed fly ashes on the durability and the corrosion of steel rebars embedded in cementmodified fly ash mortars. Cem. Concr. Compos..

